# Crystal structure of 4-(2-hy­droxy-3-meth­oxy­benzyl­amino)­benzoic acid di­methyl­formamide monosolvate monohydrate

**DOI:** 10.1107/S2056989019005103

**Published:** 2019-04-18

**Authors:** Md. Serajul Haque Faizi, Saima Kamaal, Arif Ali, Musheer Ahmad, Irina A. Golenya

**Affiliations:** a Department of Chemistry, Langat Singh College, B. R. A. Bihar University, Muzaffarpur, Bihar, 842001, India; b Department of Applied Chemistry, Faculty of Engineering & Technology, Aligarh, Muslim University, Aligarh, UP 202002, India; c National Taras Shevchenko University, Department of Chemistry, Volodymyrska, str., 64, 01601, Kyiv, Ukraine

**Keywords:** crystal structure, 2-hy­droxy-3-meth­oxy-benzaldehyde, 4-amino­benzoic acid (PABA), secondary amine, hydrogen bonding, vanillin derivative, di­methyl­formamide solvate

## Abstract

The title compound, C_15_H_15_NO_4_·C_3_H_7_NO·H_2_O, a secondary amine mol­ecule, is accompanied by one equivalent of water and one equivalent of di­methyl­formamide (DMF) as solvents. The mol­ecule is non-planar, with a C_ar­yl_—CH_2_—NH—C_ar­yl_ torsion angle of −66.3 (3)°. In the crystal, O—H⋯O and N—H⋯O hydrogen-bonding inter­actions between the amine mol­ecules and the two types of solvent mol­ecule result in the formation of a layered structure extending parallel to (010).

## Abstract 2

The title compound, C_15_H_15_NO_4_·C_3_H_7_NO·H_2_O, crystallizes with one mol­ecule of water and one mol­ecule of di­methyl­formamide (DMF) as solvate mol­ecules. The mol­ecule is non-planar, with a C_ar­yl_—CH_2_—NH—C_ar­yl_ torsion angle of −66.3 (3)°.

## Chemical context   

Vanillin and vanillin derivatives are used in food and non-food applications, in fragrances and as flavouring agents for pharmaceutical products (Hocking, 1997 ▸; Walton *et al.*, 2003 ▸). Synthetic vanillin is used as an inter­mediate in the chemical and pharmaceutical industries for the production of herbicides, anti­foaming agents and drugs, such as papaverine, l-dopa and l-methyl­dopa, as well as anti­microbial agents such as trimethoprim (Fitzgerald *et al.*, 2005 ▸), and as a bacterial co-factor involved in the synthesis of folic acid (Robinson, 1966 ▸). Another example is benzocaine, the ethyl ester of *p*-amino­benzoic acid, which is a local anaesthetic. The mechanism includes inhibiting voltage-dependent sodium channels on the nerve membrane, which results in stopping the signal propagation (Neumcke *et al.*, 1981 ▸). The title compound (1) was synthesized by reduction of reported (*E*)-4-(2-hy­droxy-3-meth­oxy­benzyl­idene­amino)­benzoic acid with sodium borohydride and crystallizes as a water and di­methyl­formamide solvate. The latter Schiff base is formed by condensation of 4-amino­benzoic acid with *o*-vanilline.
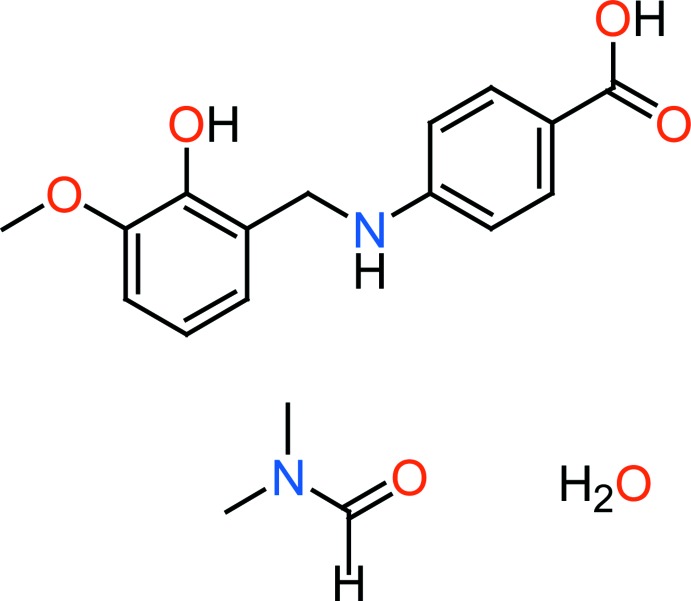


In this context and as part of an ongoing structural study of Schiff bases and secondary amines for their utilization in the synthesis of new organic compounds and the application of excited-state proton transfer and fluorescent chemosensors (Faizi *et al.*, 2016*a* ▸,*b* ▸, 2018*a* ▸,*b* ▸; Kumar *et al.*, 2018 ▸; Mukherjee *et al.*, 2018 ▸), we report here the mol­ecular and crystal structure of (1), C_15_H_15_NO_4_·C_3_H_7_NO·H_2_O.

## Structural commentary   

**Figure 1:**
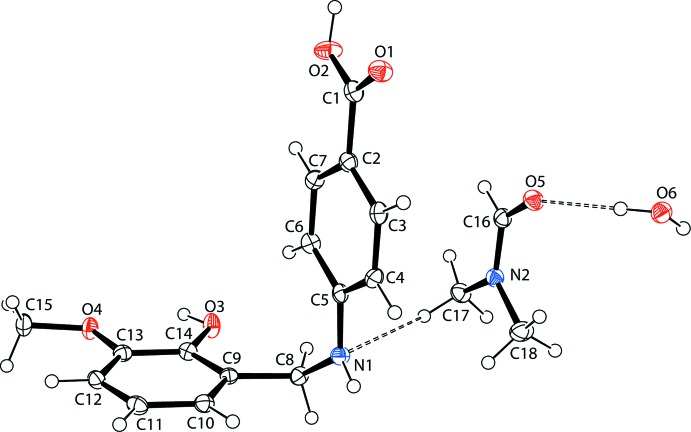
The structures of the mol­ecular entities in the asymmetric unit of the title compound. Displacement ellipsoids are drawn at the 40% probability level. Inter­molecular O—H_water_⋯O_amide_ and C—H_meth­yl_⋯N_amine_ hydrogen bonds involving the water and di­methyl­formamide solvent mol­ecules are shown as dashed lines (see Table 1 ▸ for numerical details).

Compound (1) crystallizes in space group *Pbca* with one mol­ecule of 4-(2-hy­droxy-3-meth­oxy­benzyl­amino)­benzoic acid and one mol­ecule each of DMF and water in the asymmetric unit (Fig. 1 ▸). The secondary amine has two substituted aromatic rings at either end of the —CH_2_—NH— linkage. As a result of the C_ar­yl_—CH_2_—NH— C_ar­yl_ torsion angle of −66.3 (3)°, the mol­ecular shape of the title compound is bent around the central C8—N1 bond. The secondary amine N atom (N1) has a practically trigonal-planar configuration deviating by 0.02 (1) Å from the mean plane of the adjacent atoms, and N1—C5 is apparently less conjugated with the C2–C7 benzenecarboxylic acid ring. For comparison, the reported C—N distance in the crystal structure of the ethyl 4-[(*E*)-(4-hy­droxy-3-meth­oxy­benzyl­idene)amino]­benzoate Schiff base is 1.274 (2) Å (Ling *et al.*, 2016 ▸), and in the zwitterionic form it is 1.312 Å (Kamaal *et al.*, 2018 ▸). The benzene rings C2–C7 and C9–C14 are roughly perpendicular to each another, with a dihedral angle of 88.15 (10)° between them.

The C16=O5 bond length in the dimethlyformamide solvent is 1.246 (2) Å, which is slightly longer than reported [1.2309 (17) Å (Fernandes *et al.*, 2007 ▸) or 1.2373 (18) Å (Elgemeie *et al.*, 2015 ▸)] for other di­methyl­formamide solvates. In (1), the C13—O4 bond length to the meth­oxy group is 1.366 (2) Å.

## Supra­molecular features   

**Figure 2:**
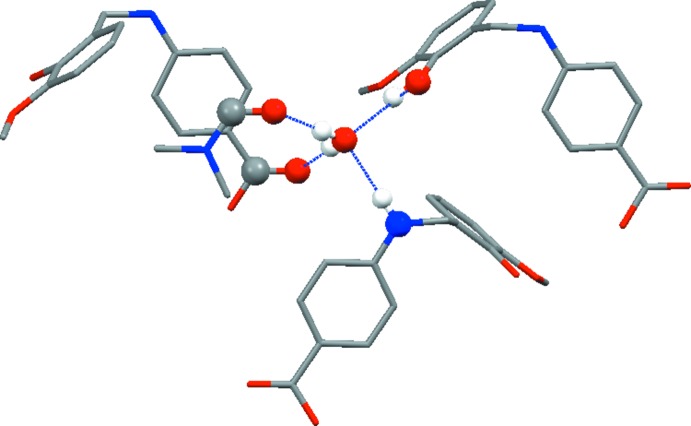
A view of hydrogen-bonding inter­actions around the water mol­ecule in the title structure.

**Figure 3:**
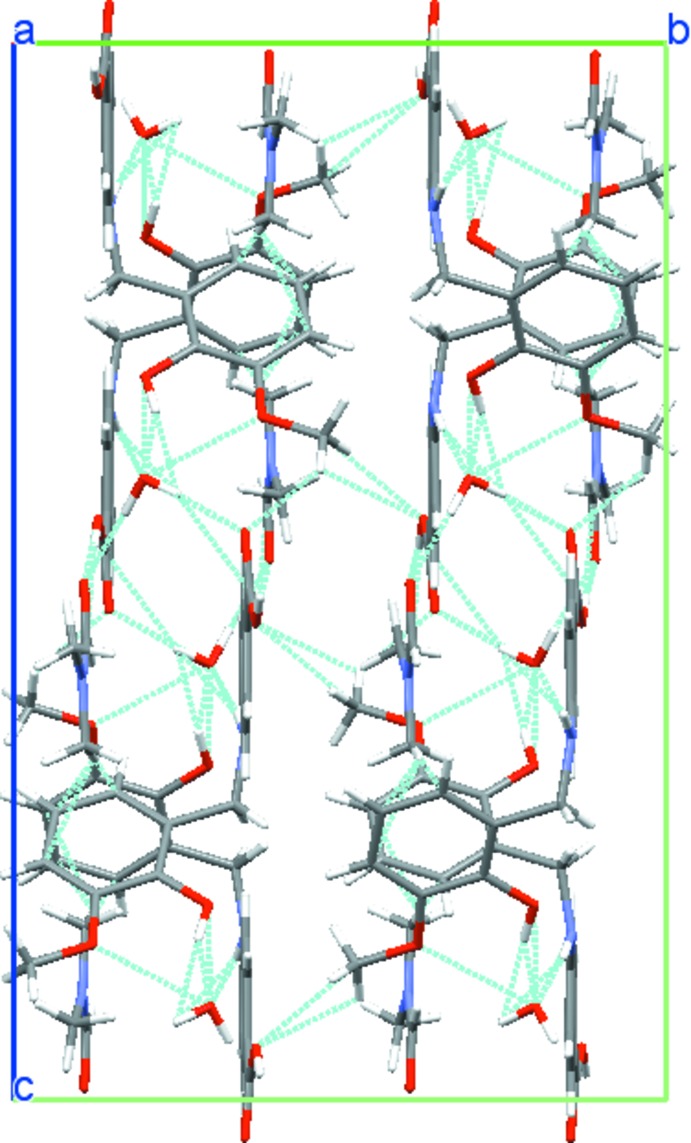
A partial view of the title structure projected along the *a* axis to emphasize the crystal packing. Dashed lines indicate hydrogen bonds (see Table 1 ▸ for numerical details).

The water and di­methyl­formamide solvent mol­ecules stabilize the packing within the crystal structure through hydrogen bonding. The molecules of di­methyl­formamide, 4-(2-hy­droxy-3-meth­oxy­benzyl­amino)­benzoic acid and water are linked through _hy­droxy_O3—H3⋯O6_water_, _amine_N1—H1⋯O6_water_, _water_O6—H6*B*⋯O5_amide_, _water_O6—H6*B*⋯O1_carb­oxy­ate_ and O2—H2⋯O5_amide_ hydrogen bonds (Table 1 ▸, Fig. 2 ▸) into a layered structure extending parallel to (010) (Fig. 3 ▸). Further C—H⋯O inter­actions (Table 1 ▸) between the methyl group of the meth­oxy functionality and the carboxyl­ate group consolidate the packing.

## Database survey   

A search of the Cambridge Structural Database (CSD, version 5.39; Groom *et al.*, 2016 ▸) gave eleven hits for reduced Schiff bases containing a C_ar­yl_—CH_2_—NH— C_ar­yl_ moiety. In direct comparison with the title compound, there are two examples of very similar compounds reported in the literature: ethyl 4-{[(2-hy­droxy­phen­yl)meth­yl]amino}­benzoate, (**I**) (WEFQEG; Salman *et al.*, 2017 ▸), and ethyl 4-[(3,5-di-*tert*-butyl-2-hy­droxy­benz­yl) amino]­benzoate, (**II**) (VABTAV; Shakir *et al.*, 2010 ▸). There is also a related compound, *viz.* ethyl 4-[(2-hy­droxy­benz­yl)amino]­benzoate, in which the 3-meth­oxy group in the title compound is replaced by a hydrogen atom and the carb­oxy­lic acid is replaced by an ester. Other related structures based on a benzyl­idene–phen­yl–amine moiety are *n*-propyl 4-[2-(4,6-di­meth­oxy­pyrimidin-2-yl­oxy)benzyl­amino]­benzoate, (**III**) (ILAGIL; Wu *et al.*, 2003 ▸), and [4-(2-hy­droxy­benzyl­amino)­benzoato-κ*O*]tri­phenyl­tin(IV), (**IV**) (WENXAP; Jiang *et al.*, 2006 ▸). The torsion angle C_ar­yl_—CH_2_—NH—C_ar­yl_ in the title compound [−66.3 (3)°] compares well to those in **I** (73.68°), **II** (77.38°) and **IV** (−87.28°), despite the difference in substituent groups.

## Synthesis and crystallization   

To a hot stirred solution of 4-amino­benzoic acid (PABA) (1.00 g, 7.2 mmol) in methanol (15 ml) was added vanillin (1.11 g, 7.2 mmol). The resultant mixture was then heated under reflux. After an hour, a precipitate was formed. The reaction mixture was heated for about a further 30 minutes for completion of the reaction, which was monitored through TLC. The reaction mixture was then cooled to room temperature, filtered and washed with hot methanol. It was then dried *in vacuo* to give (*E*)-4-(2-hy­droxy-3-meth­oxy­benzyl­idene­amino) benzoic acid in 78% yield. The latter (1.00 g, 3.7 mmol) was dissolved in 25 ml of methanol and reduced by addition of excess sodium borohydride (0.28 g, 7.4 mmol). The solution was stirred until the yellow colour disappeared. Then the solution was diluted with 8–10 times the volume of water and the pH was adjusted to 6 by addition of 12%_wt_ HCl. The white precipitate was collected and dried in air. Colourless single crystals of the title compound, suitable for X-ray analysis, were obtained by slow evaporation of a di­methyl­formamide solution.

## Refinement   

Crystal data, data collection and structure refinement details are summarized in Table 2 ▸. The N—H and O—H hydrogen atoms were located in difference-Fourier maps and were freely refined, while the C-bound H atoms were included in calculated positions and treated as riding, with fixed C—H = 0.93 Å, and *U*_iso_(H) = 1.2*U*_eq_(C,N).

## Supplementary Material

Crystal structure: contains datablock(s) I. DOI: 10.1107/S2056989019005103/wm5490sup1.cif

Structure factors: contains datablock(s) I. DOI: 10.1107/S2056989019005103/wm5490Isup2.hkl

Supporting information file. DOI: 10.1107/S2056989019005103/wm5490Isup3.cml

CCDC reference: 1909944

Additional supporting information:  crystallographic information; 3D view; checkCIF report

## Figures and Tables

**Table 1:** Hydrogen-bond geometry (Å, °)

*D*—H⋯*A*	*D*—H	H⋯*A*	*D*⋯*A*	*D*—H⋯*A*
C15—H15*C*⋯O1^i^	0.96	2.50	3.329 (3)	145
N1—H1⋯O6^iii^	0.89 (2)	2.05 (2)	2.936 (2)	175 (2)
O2—H2⋯O5^iv^	0.90 (3)	1.70 (3)	2.591 (2)	171 (3)
O3—H3⋯O6^v^	0.88 (3)	1.90 (3)	2.739 (2)	158 (3)
O6—H6*A*⋯O1^vi^	0.85 (3)	1.94 (3)	2.776 (2)	172 (3)
O6—H6*B*⋯O5	0.88 (3)	1.91 (3)	2.785 (2)	178 (3)

Symmetry codes: (i) 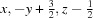; (ii) 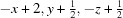; (iii) 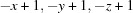; (iv) 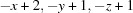; (v) 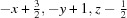; (vi) 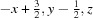.

**Table 2:** Experimental details

Crystal data	
Chemical formula	C_15_H_15_NO_4_·C_3_H_7_NO·H_2_O
*M*_r_	364.39
Crystal system, space group	Orthorhombic, *P* *b* *c* *a*
Temperature (K)	100
*a*, *b*, *c* (Å)	11.5504 (7), 13.8047 (7), 22.3899 (12)
*V* (Å^3^)	3570.1 (3)
*Z*	8
Radiation type	Mo *K*α
μ (mm^−1^)	0.10
Crystal size (mm)	0.39 × 0.24 × 0.17

Data collection	
Diffractometer	Bruker APEXII CCD
Absorption correction	–
No. of measured, independent and observed [*I* > 2σ(*I*)] reflections	40928, 3165, 2321
*R*_int_	0.106
(sin θ/λ)_max_ (Å^−1^)	0.596

Refinement	
*R*[*F* ^2^ > 2σ(*F* ^2^)], *wR*(*F* ^2^), *S*	0.044, 0.113, 1.05
No. of reflections	3165
No. of parameters	258
H-atom treatment	H atoms treated by a mixture of independent and constrained refinement
Δρ_max_, Δρ_min_ (e Å^−3^)	0.25, −0.25

Computer programs: *APEX2* (Bruker, 2014 ▸), *SAINT* (Bruker, 2014 ▸), *SHELXT2015* (Sheldrick, 2015*a* ▸), *SHELXL2016* (Sheldrick, 2015*b* ▸), *ORTEP-3 for Windows* (Farrugia, 2012 ▸), *WinGX* (Farrugia, 2012 ▸).

## supplementary crystallographic information

### Crystal data

**Table:**

C_15_H_15_NO_4_·C_3_H_7_NO·H_2_O	*D*_x_ = 1.356 Mg m^−^^3^
*M**_r_* = 364.39	Mo *K*α radiation, λ = 0.71073 Å
Orthorhombic, *P**b**c**a*	Cell parameters from 6409 reflections
*a* = 11.5504 (7) Å	θ = 2.3–28.2°
*b* = 13.8047 (7) Å	µ = 0.10 mm^−^^1^
*c* = 22.3899 (12) Å	*T* = 100 K
*V* = 3570.1 (3) Å^3^	Block, colorless
*Z* = 8	0.39 × 0.24 × 0.17 mm
*F*(000) = 1552	

### Data collection

**Table:**

Bruker APEXII CCD diffractometer	*R*_int_ = 0.106
φ and ω scans	θ_max_ = 25.1°, θ_min_ = 2.9°
40928 measured reflections	*h* = −13→13
3165 independent reflections	*k* = −16→16
2321 reflections with *I* > 2σ(*I*)	*l* = −26→26

### Refinement

**Table:**

Refinement on *F*^2^	0 restraints
Least-squares matrix: full	Hydrogen site location: mixed
*R*[*F*^2^ > 2σ(*F*^2^)] = 0.044	H atoms treated by a mixture of independent and constrained refinement
*wR*(*F*^2^) = 0.113	*w* = 1/[σ^2^(*F*_o_^2^) + (0.0393*P*)^2^ + 2.7178*P*] where *P* = (*F*_o_^2^ + 2*F*_c_^2^)/3
*S* = 1.05	(Δ/σ)_max_ < 0.001
3165 reflections	Δρ_max_ = 0.25 e Å^−^^3^
258 parameters	Δρ_min_ = −0.25 e Å^−^^3^

### Special details

**Table:**

Geometry. All esds (except the esd in the dihedral angle between two l.s. planes) are estimated using the full covariance matrix. The cell esds are taken into account individually in the estimation of esds in distances, angles and torsion angles; correlations between esds in cell parameters are only used when they are defined by crystal symmetry. An approximate (isotropic) treatment of cell esds is used for estimating esds involving l.s. planes.

### Fractional atomic coordinates and isotropic or equivalent isotropic displacement parameters (Å^2^)

**Table:**

	*x*	*y*	*z*	*U*_iso_*/*U*_eq_	
O1	0.97909 (13)	0.64576 (11)	0.53644 (7)	0.0253 (4)	
O2	1.07571 (14)	0.62871 (12)	0.45097 (7)	0.0283 (4)	
O3	0.71716 (14)	0.70129 (10)	0.18430 (7)	0.0252 (4)	
O4	0.76784 (13)	0.88000 (10)	0.14629 (6)	0.0225 (4)	
N1	0.56965 (16)	0.64392 (13)	0.34340 (8)	0.0202 (4)	
C1	0.97938 (19)	0.63895 (15)	0.48187 (10)	0.0203 (5)	
C2	0.87414 (18)	0.64213 (15)	0.44484 (9)	0.0191 (5)	
C3	0.76483 (19)	0.64465 (15)	0.47181 (10)	0.0205 (5)	
H3A	0.759163	0.644607	0.513245	0.025*	
C4	0.66632 (18)	0.64718 (15)	0.43802 (9)	0.0195 (5)	
H4	0.594711	0.649520	0.456958	0.023*	
C5	0.67081 (18)	0.64633 (14)	0.37540 (9)	0.0182 (5)	
C6	0.78028 (18)	0.64468 (15)	0.34804 (9)	0.0209 (5)	
H6	0.786010	0.645126	0.306608	0.025*	
C7	0.87952 (18)	0.64238 (15)	0.38257 (10)	0.0204 (5)	
H7	0.951416	0.640972	0.363889	0.024*	
C8	0.56478 (18)	0.66082 (15)	0.27954 (9)	0.0198 (5)	
H8A	0.487059	0.646580	0.265676	0.024*	
H8B	0.617048	0.615927	0.259956	0.024*	
C9	0.59608 (18)	0.76308 (15)	0.26052 (9)	0.0183 (5)	
C10	0.54893 (18)	0.84270 (15)	0.29065 (9)	0.0204 (5)	
H10	0.499734	0.832750	0.322951	0.024*	
C11	0.57492 (18)	0.93582 (15)	0.27275 (9)	0.0216 (5)	
H11	0.543392	0.988120	0.293264	0.026*	
C12	0.64763 (18)	0.95228 (15)	0.22444 (9)	0.0204 (5)	
H12	0.664327	1.015250	0.212442	0.024*	
C13	0.69509 (18)	0.87437 (15)	0.19427 (9)	0.0184 (5)	
C14	0.66910 (18)	0.77913 (15)	0.21267 (9)	0.0185 (5)	
C15	0.8038 (2)	0.97495 (15)	0.12804 (10)	0.0246 (5)	
H15A	0.838277	1.007996	0.161321	0.037*	
H15B	0.737862	1.010846	0.114248	0.037*	
H15C	0.859436	0.969537	0.096352	0.037*	
O5	0.73401 (13)	0.39240 (11)	0.48915 (6)	0.0232 (4)	
N2	0.63766 (15)	0.39534 (12)	0.40067 (8)	0.0197 (4)	
C16	0.73235 (19)	0.38897 (15)	0.43356 (10)	0.0207 (5)	
H16	0.802632	0.381408	0.413834	0.025*	
C17	0.64116 (19)	0.39045 (16)	0.33595 (9)	0.0233 (5)	
H17A	0.718785	0.376788	0.323146	0.035*	
H17B	0.616637	0.451334	0.319537	0.035*	
H17C	0.590384	0.340011	0.322353	0.035*	
C18	0.52415 (19)	0.40815 (18)	0.42793 (10)	0.0287 (6)	
H18A	0.533133	0.419569	0.469968	0.043*	
H18B	0.478642	0.350798	0.421860	0.043*	
H18C	0.485877	0.462530	0.409914	0.043*	
O6	0.63671 (14)	0.30090 (13)	0.58799 (7)	0.0227 (4)	
H1	0.506 (2)	0.6567 (16)	0.3644 (10)	0.025 (6)*	
H2	1.138 (3)	0.623 (2)	0.4752 (13)	0.060 (10)*	
H3	0.759 (2)	0.7170 (19)	0.1528 (12)	0.045 (8)*	
H6A	0.607 (2)	0.250 (2)	0.5735 (12)	0.044 (9)*	
H6B	0.667 (2)	0.329 (2)	0.5565 (13)	0.047 (9)*	

### Atomic displacement parameters (Å^2^)

**Table:**

	*U*^11^	*U*^22^	*U*^33^	*U*^12^	*U*^13^	*U*^23^
O1	0.0240 (9)	0.0326 (9)	0.0193 (9)	0.0028 (7)	−0.0005 (7)	0.0003 (7)
O2	0.0179 (9)	0.0423 (10)	0.0247 (9)	0.0061 (7)	0.0004 (7)	−0.0053 (7)
O3	0.0359 (10)	0.0164 (8)	0.0234 (9)	0.0002 (7)	0.0095 (7)	−0.0016 (7)
O4	0.0277 (9)	0.0182 (8)	0.0217 (8)	−0.0013 (6)	0.0070 (7)	0.0019 (6)
N1	0.0190 (10)	0.0251 (10)	0.0165 (10)	0.0002 (8)	0.0014 (8)	0.0014 (8)
C1	0.0243 (12)	0.0164 (11)	0.0202 (13)	0.0024 (9)	0.0011 (9)	−0.0017 (9)
C2	0.0218 (12)	0.0156 (11)	0.0199 (12)	0.0009 (9)	−0.0004 (9)	−0.0008 (9)
C3	0.0255 (12)	0.0185 (12)	0.0175 (11)	−0.0009 (9)	0.0026 (9)	0.0010 (9)
C4	0.0178 (11)	0.0206 (12)	0.0199 (12)	−0.0018 (9)	0.0038 (9)	0.0000 (9)
C5	0.0212 (12)	0.0117 (10)	0.0216 (12)	−0.0006 (9)	0.0001 (9)	0.0005 (8)
C6	0.0254 (12)	0.0204 (12)	0.0169 (11)	0.0017 (9)	0.0019 (9)	−0.0016 (9)
C7	0.0189 (11)	0.0182 (11)	0.0240 (12)	0.0013 (9)	0.0042 (9)	−0.0020 (9)
C8	0.0195 (11)	0.0225 (12)	0.0173 (11)	−0.0023 (9)	0.0003 (9)	−0.0003 (9)
C9	0.0171 (11)	0.0192 (11)	0.0185 (11)	−0.0019 (9)	−0.0053 (9)	0.0009 (9)
C10	0.0182 (11)	0.0233 (12)	0.0196 (12)	0.0014 (9)	0.0003 (9)	0.0006 (9)
C11	0.0215 (12)	0.0192 (12)	0.0242 (12)	0.0054 (9)	−0.0002 (9)	−0.0029 (9)
C12	0.0208 (11)	0.0157 (11)	0.0246 (12)	0.0013 (9)	−0.0031 (9)	0.0011 (9)
C13	0.0175 (11)	0.0223 (12)	0.0156 (11)	0.0003 (9)	−0.0020 (9)	0.0001 (9)
C14	0.0200 (11)	0.0184 (11)	0.0170 (11)	0.0013 (9)	−0.0009 (9)	−0.0029 (9)
C15	0.0290 (13)	0.0181 (12)	0.0265 (13)	−0.0025 (10)	0.0044 (10)	0.0042 (9)
O5	0.0247 (8)	0.0262 (9)	0.0188 (8)	0.0007 (7)	−0.0020 (6)	0.0019 (7)
N2	0.0198 (10)	0.0194 (10)	0.0198 (10)	0.0007 (7)	0.0014 (8)	0.0010 (7)
C16	0.0211 (12)	0.0169 (11)	0.0241 (13)	0.0006 (9)	0.0018 (9)	0.0011 (9)
C17	0.0246 (12)	0.0266 (13)	0.0186 (12)	0.0026 (10)	−0.0003 (9)	0.0006 (9)
C18	0.0207 (12)	0.0386 (14)	0.0267 (13)	0.0027 (10)	0.0038 (10)	0.0013 (11)
O6	0.0226 (8)	0.0265 (9)	0.0190 (9)	−0.0021 (7)	0.0003 (7)	0.0005 (7)

### Geometric parameters (Å, º)

**Table:**

O1—C1	1.225 (2)	C9—C10	1.400 (3)
O2—C1	1.318 (3)	C10—C11	1.380 (3)
O2—H2	0.90 (3)	C10—H10	0.9300
O3—C14	1.366 (2)	C11—C12	1.388 (3)
O3—H3	0.88 (3)	C11—H11	0.9300
O4—C13	1.366 (2)	C12—C13	1.383 (3)
O4—C15	1.434 (2)	C12—H12	0.9300
N1—C5	1.371 (3)	C13—C14	1.410 (3)
N1—C8	1.450 (3)	C15—H15A	0.9600
N1—H1	0.89 (2)	C15—H15B	0.9600
C1—C2	1.472 (3)	C15—H15C	0.9600
C2—C7	1.396 (3)	O5—C16	1.246 (2)
C2—C3	1.400 (3)	N2—C16	1.321 (3)
C3—C4	1.367 (3)	N2—C17	1.451 (3)
C3—H3A	0.9300	N2—C18	1.457 (3)
C4—C5	1.403 (3)	C16—H16	0.9300
C4—H4	0.9300	C17—H17A	0.9600
C5—C6	1.405 (3)	C17—H17B	0.9600
C6—C7	1.383 (3)	C17—H17C	0.9600
C6—H6	0.9300	C18—H18A	0.9600
C7—H7	0.9300	C18—H18B	0.9600
C8—C9	1.518 (3)	C18—H18C	0.9600
C8—H8A	0.9700	O6—H6A	0.85 (3)
C8—H8B	0.9700	O6—H6B	0.88 (3)
C9—C14	1.382 (3)		
			
C1—O2—H2	111.3 (19)	C9—C10—H10	119.8
C14—O3—H3	113.6 (18)	C10—C11—C12	120.70 (19)
C13—O4—C15	117.01 (16)	C10—C11—H11	119.6
C5—N1—C8	122.99 (18)	C12—C11—H11	119.6
C5—N1—H1	115.1 (15)	C13—C12—C11	119.53 (19)
C8—N1—H1	117.1 (15)	C13—C12—H12	120.2
O1—C1—O2	122.3 (2)	C11—C12—H12	120.2
O1—C1—C2	123.9 (2)	O4—C13—C12	125.70 (19)
O2—C1—C2	113.87 (18)	O4—C13—C14	114.44 (17)
C7—C2—C3	118.1 (2)	C12—C13—C14	119.87 (19)
C7—C2—C1	121.73 (19)	O3—C14—C9	118.85 (18)
C3—C2—C1	120.17 (19)	O3—C14—C13	120.74 (18)
C4—C3—C2	120.8 (2)	C9—C14—C13	120.40 (19)
C4—C3—H3A	119.6	O4—C15—H15A	109.5
C2—C3—H3A	119.6	O4—C15—H15B	109.5
C3—C4—C5	121.5 (2)	H15A—C15—H15B	109.5
C3—C4—H4	119.3	O4—C15—H15C	109.5
C5—C4—H4	119.3	H15A—C15—H15C	109.5
N1—C5—C4	119.40 (19)	H15B—C15—H15C	109.5
N1—C5—C6	122.59 (19)	C16—N2—C17	122.02 (18)
C4—C5—C6	118.0 (2)	C16—N2—C18	121.30 (18)
C7—C6—C5	120.2 (2)	C17—N2—C18	116.68 (18)
C7—C6—H6	119.9	O5—C16—N2	124.5 (2)
C5—C6—H6	119.9	O5—C16—H16	117.7
C6—C7—C2	121.4 (2)	N2—C16—H16	117.7
C6—C7—H7	119.3	N2—C17—H17A	109.5
C2—C7—H7	119.3	N2—C17—H17B	109.5
N1—C8—C9	114.65 (17)	H17A—C17—H17B	109.5
N1—C8—H8A	108.6	N2—C17—H17C	109.5
C9—C8—H8A	108.6	H17A—C17—H17C	109.5
N1—C8—H8B	108.6	H17B—C17—H17C	109.5
C9—C8—H8B	108.6	N2—C18—H18A	109.5
H8A—C8—H8B	107.6	N2—C18—H18B	109.5
C14—C9—C10	119.03 (19)	H18A—C18—H18B	109.5
C14—C9—C8	120.80 (19)	N2—C18—H18C	109.5
C10—C9—C8	120.15 (19)	H18A—C18—H18C	109.5
C11—C10—C9	120.5 (2)	H18B—C18—H18C	109.5
C11—C10—H10	119.8	H6A—O6—H6B	103 (2)
			
O1—C1—C2—C7	174.4 (2)	C14—C9—C10—C11	0.1 (3)
O2—C1—C2—C7	−5.1 (3)	C8—C9—C10—C11	−178.52 (19)
O1—C1—C2—C3	−5.8 (3)	C9—C10—C11—C12	0.4 (3)
O2—C1—C2—C3	174.68 (19)	C10—C11—C12—C13	−0.5 (3)
C7—C2—C3—C4	0.1 (3)	C15—O4—C13—C12	4.2 (3)
C1—C2—C3—C4	−179.70 (19)	C15—O4—C13—C14	−175.82 (18)
C2—C3—C4—C5	0.7 (3)	C11—C12—C13—O4	−179.86 (19)
C8—N1—C5—C4	168.25 (18)	C11—C12—C13—C14	0.1 (3)
C8—N1—C5—C6	−14.0 (3)	C10—C9—C14—O3	178.43 (18)
C3—C4—C5—N1	176.67 (19)	C8—C9—C14—O3	−3.0 (3)
C3—C4—C5—C6	−1.2 (3)	C10—C9—C14—C13	−0.4 (3)
N1—C5—C6—C7	−176.78 (19)	C8—C9—C14—C13	178.20 (19)
C4—C5—C6—C7	1.0 (3)	O4—C13—C14—O3	1.5 (3)
C5—C6—C7—C2	−0.3 (3)	C12—C13—C14—O3	−178.52 (19)
C3—C2—C7—C6	−0.2 (3)	O4—C13—C14—C9	−179.71 (18)
C1—C2—C7—C6	179.53 (19)	C12—C13—C14—C9	0.3 (3)
C5—N1—C8—C9	−66.3 (3)	C17—N2—C16—O5	−179.74 (19)
N1—C8—C9—C14	135.2 (2)	C18—N2—C16—O5	0.6 (3)
N1—C8—C9—C10	−46.3 (3)		

### Hydrogen-bond geometry (Å, º)

**Table:**

*D*—H···*A*	*D*—H	H···*A*	*D*···*A*	*D*—H···*A*
C15—H15*C*···O1^i^	0.96	2.50	3.329 (3)	145
C15—H15*C*···O2^ii^	0.96	2.55	3.094 (3)	116
N1—H1···O6^iii^	0.89 (2)	2.05 (2)	2.936 (2)	175 (2)
O2—H2···O5^iv^	0.90 (3)	1.70 (3)	2.591 (2)	171 (3)
O3—H3···O6^v^	0.88 (3)	1.90 (3)	2.739 (2)	158 (3)
O6—H6*A*···O1^vi^	0.85 (3)	1.94 (3)	2.776 (2)	172 (3)
O6—H6*B*···O5	0.88 (3)	1.91 (3)	2.785 (2)	178 (3)

Symmetry codes: (i) *x*, −*y*+3/2, *z*−1/2; (ii) −*x*+2, *y*+1/2, −*z*+1/2; (iii) −*x*+1, −*y*+1, −*z*+1; (iv) −*x*+2, −*y*+1, −*z*+1; (v) −*x*+3/2, −*y*+1, *z*−1/2; (vi) −*x*+3/2, *y*−1/2, *z*.

## References

[bb1] Bruker (2014). *APEX2*, *SAINT* and *SADABS*. Bruker AXS Inc., Madison, Wisconsin, USA.

[bb2] Elgemeie, G. H., Mohamed, R. A., Hussein, H. A. & Jones, P. G. (2015). *Acta Cryst.* E**71**, 1322–1324. 10.1107/S2056989015018903 PMC4645039 26594501

[bb3] Faizi, M. S. H., Alam, M. J., Haque, A., Ahmad, S., Shahid, M. & Ahmad, M. (2018*a*). *J. Mol. Struct.* **1156**, 457–464.

[bb4] Faizi, M. S. H., Ali, A. & Potaskalov, V. A. (2016*a*). *Acta Cryst.* E**72**, 1366–1369. 10.1107/S205698901601344X PMC5050754 27746919

[bb5] Faizi, M. S. H., Dege, N. & Iskenderov, T. S. (2018*b*). *Acta Cryst.* E**74**, 410–413. 10.1107/S2056989018003043 PMC5947814 29765734

[bb6] Faizi, M. S. H., Gupta, S., Mohan, V. K., Jain, K. V. & Sen, P. (2016*b*). *Sens. Actuators B Chem.* **222**, 15–20.

[bb7] Farrugia, L. J. (2012). *J. Appl. Cryst.* **45**, 849–854.

[bb8] Fernandes, P., Florence, A. J., Fabbiani, F., David, W. I. F. & Shankland, K. (2007). *Acta Cryst.* E**63**, o4861. 10.1107/S1600536807067232 PMC2960435 21201387

[bb9] Fitzgerald, D. J., Stratford, M., Gasson, M. J. & Narbad, A. (2005). *J. Agric. Food Chem.* **53**, 1769–1775. 10.1021/jf048575t 15740072

[bb10] Groom, C. R., Bruno, I. J., Lightfoot, M. P. & Ward, S. C. (2016). *Acta Cryst.* B**72**, 171–179. 10.1107/S2052520616003954 PMC4822653 27048719

[bb11] Hocking, M. B. (1997). *J. Chem. Educ.* **74**, 1055–1059.

[bb12] Jiang, H., Ma, J.-F. & Zhang, W.-L. (2006). *Acta Cryst.* E**62**, m2745–m2746.

[bb13] Kamaal, S., Faizi, M. S. H., Ali, A., Ahmad, M. & Iskenderov, T. (2018). *Acta Cryst.* E**74**, 1847–1850. 10.1107/S2056989018016262 PMC6281083 30574386

[bb14] Kumar, M., Kumar, A., Faizi, M. S. H., Kumar, S., Singh, M. K., Sahu, S. K., Kishor, S. & John, R. P. (2018). *Sens. Actuators B Chem.* **260**, 888–899.

[bb15] Ling, J., Kavuru, P., Wojtas, L. & Chadwick, K. (2016). *Acta Cryst.* E**72**, 951–954. 10.1107/S2056989016008999 PMC4992913 27555938

[bb16] Mukherjee, P., Das, A., Faizi, M. S. H. & Sen, P. (2018). *Chemistry Select*, **3**, 3787–3796.

[bb17] Neumcke, B., Schwarz, W. & Stampfli, R. (1981). *Pflugers Arch.* **390**, 230–236. 10.1007/BF00658267 6265861

[bb18] Robinson, F. A. (1966). *The Vitamin Co-factors of Enzyme Systems*, pp. 541–662 London: Pergamon.

[bb19] Salman, M., Abu-Yamin, A. A., Sarairah, I., Ibrahim, A. & Aldamen, M. A. (2017). *Z. Kristallogr.* **232**, 631–632.

[bb20] Shakir, R. M., Ariffin, A. & Ng, S. W. (2010). *Acta Cryst.* E**66**, o2916. 10.1107/S1600536810040742 PMC3009098 21589090

[bb21] Sheldrick, G. M. (2015*a*). *Acta Cryst.* A**71**, 3–8.

[bb22] Sheldrick, G. M. (2015*b*). *Acta Cryst.* C**71**, 3–8.

[bb23] Walton, N. J., Mayer, M. J. & Narbad, A. (2003). *Phytochemistry*, **63**, 505–515. 10.1016/s0031-9422(03)00149-3 12809710

[bb24] Wu, J., Zhang, P.-Z., Lu, L., Yu, Q.-S., Hu, X.-R. & Gu, J.-M. (2003). *Chin. J. Struct. Chem.* **22**, 613–616.

